# Ozone acting on human blood yields a hormetic dose-response relationship

**DOI:** 10.1186/1479-5876-9-66

**Published:** 2011-05-17

**Authors:** Velio A Bocci, Iacopo Zanardi, Valter Travagli

**Affiliations:** 1 Dipartimento di Fisiologia, Università degli Studi di Siena, Viale Aldo Moro, 2, 53100, Siena, Italy; 2 Dipartimento Farmaco Chimico Tecnologico and European Research Center for Drug Discovery and Development, Università degli Studi di Siena, Viale Aldo Moro, 2, 53100, Siena, Italy

## Abstract

The aim of this paper is to analyze why ozone can be medically useful when it dissolves in blood or in other biological fluids. In reviewing a number of clinical studies performed in Peripheral Arterial Diseases (PAD) during the last decades, it has been possible to confirm the long-held view that the inverted U-shaped curve, typical of the hormesis concept, is suitable to represent the therapeutic activity exerted by the so-called ozonated autohemotherapy. The quantitative and qualitative aspects of human blood ozonation have been also critically reviewed in regard to the biological, therapeutic and safety of ozone. It is hoped that this gas, although toxic for the pulmonary system during prolonged inhalation, will be soon recognized as a useful agent in oxidative-stress related diseases, joining other medical gases recently thought to be of therapeutic importance. Finally, the elucidation of the mechanisms of action of ozone as well as the obtained results in PAD may encourage clinical scientists to evaluate ozone therapy in vascular diseases in comparison to the current therapies.

## Introduction

Ozone is a double-faceted gas. It has a crucial protective relevance in partially blocking mutagenic and carcinogenic UV radiations emitted by the sun (wavelengths of 100-280 nm) in the stratosphere [1], while its increasing concentration in the troposphere causes severe pulmonary damage and increased mortality [2,3]. In spite of this drawback, there are growing experimental and clinical evidences about the medical use of ozone [4-11]. Since XVI Century, Paracelsus had ingeniously guessed that "all things are poison and nothing is without poison and only the right dose differentiates a poison from a remedy". In 2005, Timbrell reiterated the concept in his book: "The poison paradox; chemicals as friends and foes" [12]. During the Earth evolution, harnessing oxygen by metazoans has allowed a fantastic biodiversity and growth but it has also created a slow acting "poison". It is reasonable to believe that the antioxidant system slowly evolved and specialized during the last two billion years for counteracting the daily formation (3-5 g in humans) of anion superoxide in the mitochondria and the release of H_2_O_2 _by ubiquitous NADPH oxidases. However, there is a general consensus that the physiological production of H_2_O_2 _is essential for life. Olivieri *et al. *[13] and Wolff [14] were the first to describe the effect of either low concentrations of radioactive thymidine or of a very low dose of radiation inducing an adaptive response in human cells in comparison to a high dose. Goldman [15] introduced the term "hormesis" to mean "the beneficial effect of a low level exposure to an agent that is harmful at high levels". It goes to the merit of Calabrese [16-19] to have experimentally controlled this concept and to have presented a number of examples of stimulatory responses following stimuli below the toxicological threshold. Until 2002 ozone therapy was pharmacologically conceived as a therapy where low ozone doses were stimulatory, while high doses were inhibitory. This conception, reflecting the classical idea that a low antigen dose is stimulatory, where an antigen overdose is inhibitory, was vague and unsuitable because ozone acts in a complex way and a high dose can still be effective but accompanied by side-effects. Indeed, one of us in 2002 amply delineated the sequence of biochemical reactions elicited *ex vivo *after the addition of a certain volume of O_2_-O_3 _gas mixture to an equal volume of human blood [20]. First of all, mixing blood with an oxidant implies a calculated and precise oxidative stress, i.e. a homeostatic change with production of highly reactive messengers. The oxidative stress, like many others, induces a biological response leading to an adaptive phenomenon. The teleological significance of this response is universal, from bacteria to plants and Mammals, and small repetitive stresses induce an extremely useful adaption response represented by the revival of critical defense mechanisms [20-22]. At the same time, Calabrese and Baldwin described the "overcompensation stimulation hormesis" (OCSH) as the result of a compensatory biological process following an initial disruption in homeostasis [17]. After a reviewer's information also Re later on had expressed this possibility [23]. Ozone presents some subtle differences that will be explained by clarifying the biochemical reactions occurring between the organic compounds of plasma and this gas.

## Ozone is a Strong Oxidant Gas

The three oxygen atoms in gas-phase ozone form an isosceles triangle with a distance among the equal sides of 1.26 Å, and exist in several mesomeric states in dynamic equilibrium [24]. In terms of oxidation potential (E°), ozone (2.07 V) is the third after fluorine (3.06 V) and hydroxyl radical (2.80 V). Other pertinent oxidants are: hydrogen peroxide (1.77 V), hypochlorous acid (1.49 V) and chlorine (1.36 V). Ozone has a paired number of electrons in the external orbit and, although it is not a radical molecule, it is far more reactive than oxygen and readily generates some of the ROS produced by oxygen. Ozone is very unstable and at 20 °C, with a half-life of about 40 min, it decomposes according to the exothermic reaction:

Such an aspect has generated the idea that ozone will donate its energy to the organism by reacting with specific body compartments [20]. However, after having ascertained the complexity of the mechanism of action, the conclusion is that ozone dissolved in the water of plasma acts as a pro-drug, generating chemical messengers which will accelerate transfer of electrons and the overall metabolism. It goes to the merit of Hans Wolff (1927-1980), a German physician, to have developed the O_3_-AHT by insufflating *ex vivo *a gas mixture composed of medical oxygen (95%) and ozone (5%) into the blood contained in a dispensable ozone-resistant and sterile glass bottle [25].

## Which are the Blood Components Reacting with Ozone?

**Figure 1:**
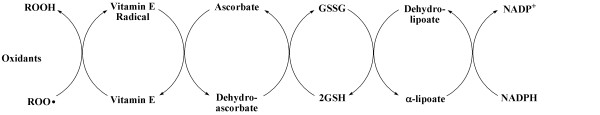
**Cellular responses to oxidant exposure**. ROOH and ROO• indicate lipohydroperoxide and its oxygen centered organic radicals formed by radical reactions with cellular components, respectively. GSH and GSSG represent the sulfhydryl/disulfide pair of glutathione species. Nicotinamide adenine dinucleotide phosphate, NADP(H), is the primary electron source, regenerated by the cellular reduction systems.

For almost three decades ozone therapy was used only in Germany by practitioners who, by using empirical procedures, elicited skepticism and prejudice in academic clinical scientists. Only during the last fifteen years, by using modern ozone generators able to photometrically (253.7 nm) measure the ozone concentration in a specified gas volume, in real time, and in a precise manner (hence the precise ozone dose per ml of blood), it has been possible to accurately study the reactions of ozone with human blood. It has been clarified that ozone toxicity depends upon its dose and, more important, that judicious ozone dosages can be neutralized by biological defenses [4,20-22,26]. Blood contains some 55% of plasma and about 45% of cells, the bulk of which is represented by erythrocytes. The composition of plasma is complex but, simply said, it contains: about 92% of water; dissolved ions such as HCO_3_^- ^and PO_4_^3- ^regulate the pH within the range of 7.3-7.4; both hydrophilic (glucose, uric acid, ascorbic acid, cysteine and other amino acids) and lipophilic (bilirubin, vitamin E, carotenoids, lycopene) molecules; about 5 mg lipids (triglycerides, cholesterol, phospholipids and lipoproteins); proteins, among which albumin (4.5 g/dl), fibrinogen as well as globulins, among which either transferrin or ceruloplasmin binds either Fe^2+ ^or Cu^+^, respectively, coagulation factors and hormones. Among the plasma main functions, one is the antioxidant activity performed by a variety of molecules such as uric acid (4.0-7.0 mg/dl, 400 μM), ascorbic acid (Aa) (0.4 - 1.5 mg/dl, 22,7-85 µ;M), GSH (0.5-1.0 μM), the mentioned lipophilic compounds as well as albumin. In detail, erythrocytes have a great reservoir of GSH (about 1 mmol/l), thioredoxin with two available cysteine, and potent antioxidant enzymes (catalase, GSH-Rd, GSH-Px, GSH-Tr, and SOD). They can quickly wipe out great amounts of oxidants such as ^·^OH, H_2_O_2_, OCl^-^, ONOO^- ^and, at the same time, recycle protons back to oxidised compounds by using protons donated by NADPH continuously regenerated by the activity of G6PD via the pentose phosphate pathway. It must be noted that most of these antioxidants work in concert accelerating the reduction of noxious oxidants (Figure 1). Albumin on its own is the most important because it holds nucleophilic residues, such as one free Cys34 as well as multiple Lys199 and His146 [27,28].

## The Biochemical Reactions of Ozone with Blood

During the most precise and safe methodological *ex vivo *O_3_-AHT approach, oxygen-ozone mixture dissolves into the water of plasma. Oxygen has a low solubility, but the pO_2 _slowly raises up to about 400 mmHg [29]. Hemoglobin become fully oxygenated (Hb_4_O_8_) but this is hardly relevant because, during the infusion period, it mixes with venous blood which has a pO_2 _of about 40 mmHg. On the other hand, ozone behaves quite differently because, by immediately reacting with ions and biomolecules, it does not follow the classical Henry's law in terms of linear solubility variation with pressure. First of all ozone is about tenfold more soluble than oxygen and, as ozone dissolves in the plasmatic water, it instantaneously reacts with hydrophilic antioxidants: by using an ozone concentration of 40 μg/ml, corresponding to 0.84 µ;mol/ml per ml of blood, within five min an average of 78% of Aa has been oxidized to dehydroascorbate and about 20% of uric acid has been oxidized to allantoin [30]. Only about 10% of alpha tocopherol has formed an alpha tocopheryl radical. At the same time the remaining ozone performs the peroxidation of available unsaturated fatty acids, which represent an elective substrate and are mostly albumin-bound. Peroxidation of n-6 PUFA leads to the formation of H_2_O_2 _and 4-hydroxy-2E-nonenal (4-HNE) [31], while n-3 PUFA leads to the formation of 4-hydroxy-2E-hexenal (4-HHE) [32,33]:

As all of these reactions happen in a few seconds, ozone, until present in the gas phase, continues to dissolve in the plasmatic water and instantly reacts. Within the canonical 5 min, ozone is fully extinct with both a rather small depletion of hydrosoluble antioxidants and the simultaneous plasmatic increase of ROS and LOP. The ozonated blood is then infused into the donor patient.

## What is the Significance and Fate of These Ozone Messengers?

First of all the brief life-span of H_2_O_2 _will be discussed. During the 5 min of mixing blood with the gas *ex vivo*, H_2_O_2 _will dynamically increase its concentration: rapid at first and progressively slowing down as ozone is being depleted. With the therapeutically high ozone concentration of 80 μg/ml per ml blood, the H_2_O_2 _concentration measured in plasma after 2.5 min is at most 40 μM because the rate of synthesis is equilibrated by multiple degradation routes. Some H_2_O_2 _is reduced by free soluble antioxidants including traces of catalase and GSH-Px. As the hemolysis is negligible (<0.5%), free Fe^2+ ^or Cu^+ ^are not present and it is unlikely that hydroxyl ions are ever formed by either the Fenton-Jackson or the Haber-Weiss reactions. As H_2_O_2 _is unionized, it freely diffuse into all blood cells although the bulk is mopped up by erythrocytes. The establishment of a dynamic, yet transitory, H_2_O_2 _gradient between the plasma and the cytoplasmatic water of blood cells makes this oxidant a very early effector. Its final intracellular concentration may be not higher than 10%, hence 3-4 μmoles, as it has been demonstrated in other studies [34-39]. The smartness of this system is that the H_2_O_2 _concentration, though small, is enough to trigger several crucial biochemical reactions without toxicity because the internal cell environment contains a wealth of GSH, thioredoxin, catalase and GSH-Px, which do not allow a dangerous increase. In spite of a threshold of only a few micromoles, it has a critical relevance and means that an ozone amount below 0.42 μmol for each ml volume of the gas mixture (medical grade O_2 _≥95% and O_3 _≤5%) reacting in a 1:1 ratio with autologous blood may be ineffective, resulting in a therapeutic failure of O_3_-AHT. It is also necessary to remind that the ozonation process greatly differs whether it occurs either in plasma or in blood. In plasma, TAS levels was, as expected, ozone-dose dependent and decreased between 46 and 63% in relation to ozone concentrations of either 0.84 μmol/ml or 1.68 μmol/ml per ml of plasma, respectively. On the other hand, in blood taken from the same donors, after being treated with the same ozone concentrations, TAS only decreased from 11 to 33% in the first minute after ozonation, respectively. Moreover, it was surprising to determine that they both recovered and returned to the original value within 20 min, indicating the capacity of blood cells to quickly regenerate dehydroascorbate and GSH disulfide [34]. It has been also brilliantly demonstrated that, thanks to erythrocytes, dehydroascorbate was recycled back to Aa within 3 min [40]. On the same way, only about 20% of the intraerythrocytic GSH had been oxidized to GSSG within one min after ozonation and promptly reduced to normal after 20 min [41]. Aa, alpha-tocopherol, GSH and lipoic acid undergo an orderly reduction by a cooperative sequence of electron donation continuously supplied by NADPH-reducing equivalents to GSH-Rd and thioredoxin reductase [42] (Figure 1). These data, by showing that the therapeutic ozonation only temporarily and reversibly modifies the cellular redox homeostasis were reassuring regarding the safety of ozone as a medical drug. In summary, the initial disruption of homeostasis due to ozone oxidation is followed by the rapid reestablishment of homeostasis with two main advantages: the first being the value of triggering several biochemical reactions in blood cells and the second mediated by LOP compounds, the induction of an adaptive process due to the up-regulation of the antioxidant enzymes. This is in line with the temporal sequence of the OCSH dose-response relationship.

## What is the Action of Ozone in the Blood Cells?

### - Erythrocytes

Probably the activation of phosphofructokinase accelerates glycolysis with a demonstrated increase of ATP and 2,3-DPG [4,20]. Functionally, the oxyhemoglobin sigmoid curve shifts to the right owing to the Bohr effect, i.e. a small pH reduction (about 7.25) and a slight increase of 2,3-DPG. This metabolite increases only in patients who have a very low level but it remains to be clarified how the phosphoglyceromutase is activated. The shift to the right is advantageous for improving tissue oxygenation as the chemical bonding of oxygen to hemoglobin is attenuated, facilitating oxygen extraction from ischemic tissues. Rokitansky *et al.*, had previously shown that the pO_2 _was lowered to 20-25 mm Hg in the femoral vein of PAD's patient throughout O_3_-AHT sessions [43]. It seems obvious that erythrocytes ozonated *ex vivo *may be modified only for a brief period. Only repeated therapeutic sessions may allow to LOP compounds to reach the bone-marrow and activate a subtle development at the erythropoietic level, favouring the formation of new erythrocytes with improved biochemical characteristics, which provisionally were named "supergifted erythrocytes" [20]. If this hypothesis is correct, every day, during prolonged ozonetherapy, the bone marrow may release a cohort (about 0.9% of the pool) of new erythrocytes with improved biochemical characteristics. In fact, the therapeutic advantage does not abruptly stop with the cessation of the therapy but rather persists for 2-3 months, probably in relation to the life-span of the circulating supergifted erythrocytes [26]. It is interesting that during prolonged ozonetherapy, by isolating through a sedimentation gradient the small portion of very young erythrocytes, it has been demonstrated that they have a significant higher content of G6PD [44]. Such a result strengthens the postulation that only a cycle of more than 15 treatments (not less than 3 liters of ozonated blood) could improve an ischemic pathology.

### - Leukocytes

Human neutrophils are able to generate an ozone-like molecule [45] and volatile compounds [46] as a part of their phagocyte activity. Neutrophil phagocytic activity has been found enhanced during ozonetherapy [47]. Moreover, H_2_O_2 _activates a tyrosin-kinase with subsequent phosphorylation of IkB, one of the trimeric components at rest of the ubiquitous transcription factor denominated NF-kB [48,49]. The phosphorylated IkB detaches from the trimer and it is broken down in the proteasome. The remaining eterodimer p50-p65 is transferred into the nucleus, where it can activate about 100 genes up-regulating the synthesis of acute-phase proteins, several proinflammatory cytokines (IFN-γ, TNF-α, IL-8) and even HIV proteins [50]. There is no doubt that H_2_O_2 _is the trigger as the activation is related to a cysteine oxidation that can be prevented by an excess of thiol. Although ozone is a very modest inducer of some cytokines [50], the consequent immunomodulatory effect may be useful in immune-depressed patients after chemotherapy, or in chronic infectious diseases. It must be clear that ozone in itself cannot exist in the circulation and moreover, due to the potent antioxidant capacity of plasma, it is unable to kill any pathogens *in vivo *whereas an activated immune system may be helpful [51].

### - Platelets

During O_3_-AHT, the detection of PDGF-B, TGF-β_1_, IL-8 and EGF released in heparinized plasma in ozone- dose dependent quantities was not surprising because platelets are exquisitely sensitive to a progressive acute oxidative stress [20,52]. The increased level of these growth factors in the circulation may have the beneficial effect of enhancing the healing of foot-related problems from diabetes or PAD.

## The pleiotropic LOP activities

**Figure 2:**
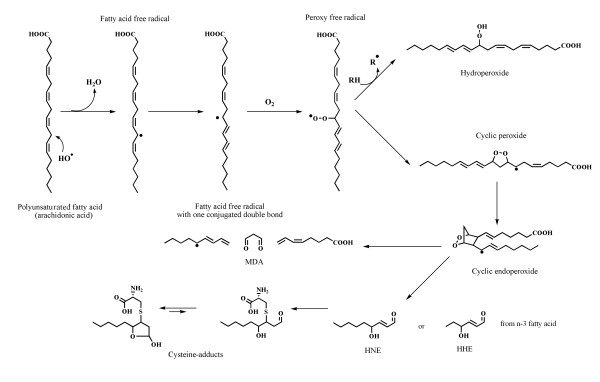
**Generic scheme of polyunsaturated fatty acids peroxidation**. Arachidonic acid reactions have been detailed, but similar pathways are applicable to other polyenoic fatty acids. MDA: malondialdehyde. HHE: 4-hydroxy-2E-hexenal. HNE: 4-hydroxy-2E-nonenal.

As shown in Figure 2, LOP production follows peroxidation of PUFA present in the plasma: they are heterogeneous and can be classified as lipoperoxide radicals, alkoxyl radicals, lipohydroperoxides, F_2_-isoprostanes, as well as aldehydes like acrolein, MDA and terminal hydroxyl alkenals, among which 4-HNE and 4-HHE. As free radicals and aldehydes are intrinsically deleterious, only precise and appropriate ozone doses must be used in order to generate them in very low concentrations. Among the aldehydes, 4-HNE is quantitatively the most important. It is an amphipathic molecule and it has a brief-half-life in saline solution. On the other hand it reacts with a variety of compounds such as albumin, enzymes, GSH, carnosine, and phospholipids [31,53]. There is no receptor for 4-HNE but it has been reported that, in concentration above 1 μM *in vitro*, after binding to more than 70 biochemical targets, it exerts some deleterious activity [31]. On the other hand, during the rapid reaction of ozone with blood, the generated hydroxy-alkenals, will form adducts both with GSH or with the abundant albumin molecules. This possibility is supported by findings which have shown that human albumin, rich in accessible nucleophilic residues, can quench up to nine 4-HNE molecules, the first being Cys34, followed by Lys199 and His146 [27,28]. Interestingly, when samples of ozonated human plasma were incubated at 37 °C for 9 hours, 4-HNE, most likely bound to albumin, remained stable [54]. These data clarify why a judicious *ex vivo *ozonation of blood does not harm the vascular system during the infusion into the donor. Aerobic organisms, in order to tolerate the continuous generation of aldehydic compounds have developed detoxifying systems as follows: the first is the ***dilution ***of these products in both the plasma and the extracellular fluid involving a volume of about 11 L in humans. The second is the ***detoxification ***operated by aldehyde dehydrogenase, aldose reductase and GSH-Tr [55,56] and the third is the ***excretion ***via bile and urine excretion [57-59]. The relevance of these catabolic pathways was appreciated when the half-life of infused alkenals present in ozonated blood in a patient was less than 5 min [60]. The interesting aspect is that albumin can transport 4-HNE in all body tissues, from liver to endocrine glands and the CNS. 4-HNE-Cys adducts, released at many sites, inform a variety of cells of a transient, acute oxidative stress and represent an important biochemical trigger. At submicromolar or picomolar levels, 4-HNE can act as a signaling molecule capable of activating the synthesis of γ-glutamate cysteine ligase, γ -glutamyl transferase, γ -glutamyl transpeptidase, HSP-70, HO-1, and antioxidant enzymes such as SOD, GSH-Px, catalase and last but not least, G6PDH, a critical electron-donor enzyme during erythropoiesis in the bone marrow. There is a wide consensus on the relevance of the induction of protective molecules during small but repeated oxidative stress [20,61-65]. In other words, the concept that a precisely controlled oxidative stress can strengthen the antioxidant defenses is well accepted today. Once again, the low level of stress by enhancing the fitness of the defense system, is consistent with the hormetic concept. Moreover at the time of ozonated blood infusion, 4-HNE-Cys adduct can also act on the vast expanse of endothelial cells and enhance the production of NO [35]. Such a crucial mediator on its own or as a nitrosothiol, with a trace of CO released with bilirubin *via *HO-1 activity, allows vasodilation, thus improving tissue oxygenation in ischemic tissues [66]. H_2_S is another potentially toxic molecule that, when released in trace amounts, it becomes an important physiological vasodilator like NO and CO [67,68]. Moreover, as it happens for the mentioned physiological traces of other gases, the small amount of ozone necessary to trigger useful biological effects is in line with the concept of the hormesis theory [69].

Another interesting aspect observed in about 2/3 of patients is a sense of wellness and physical energy throughout the ozonetherapy [70]. It is not yet known whether these feelings are due to the power of the generated ozone messengers which can modify or improve the hormonal secretion. On the other hand, the feeling of euphoria may be due to improved oxygenation or/and enhanced secretion of growth hormone, ACTH-cortisol and dehydroepiandrosterone [26,71]. Furthermore, when LOP reach the hypothalamic area they may improve the release of serotonin and endorphins, as it was observed after intense dynamic exercise [72]. Experience acquired after thousands O_3_-AHT has clarified that there is neither objective nor subjective toxicity, or to use Calabrese's acronyms, there is no observable adverse effects (NOAEL). Moreover, neither structural nor enzymatic damages have been observed in blood components after ozonation of blood within the therapeutic window [73,74]. On the other hand, patients with more advanced disease during the initial session especially if performed with a high ozone dosage, frequently report to feel very tired and sleepy. This is the lowest observed adverse effect level (LOAEL) that has been observed in about 10% of PAD's patients with stage III and IV of the Leriche-Fontaine's classification. Such a knowledge compels to begin always with low ozone dosage and carefully observe the patient's response.

## Which is the Most Suitable Term for Describing the Dose-Response Relationship Between Ozone and Blood?

**Figure 3:**
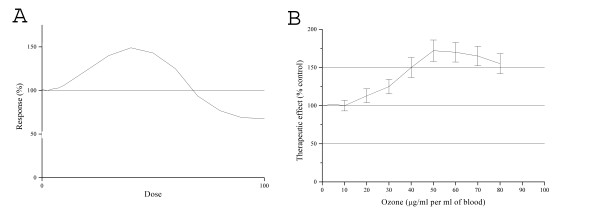
**The hypothetical inverted U-shaped curve describing an ideal dose-response relationship (panel A)**. The inverted U-shaped curve drawn on the basis of the therapeutic effect in PAD's patients by using an ozone concentration range between 15 and 80 μg/ml of gas per ml of blood. During a course of 15-20 sessions, the initial ozone concentration of 10 μg/ml has been slowly upgraded to the concentration of 80 μg/ml (panel B). The end-points that have been considered to determine the therapeutic effects are: claudication; ankle-brachial index; disappearance of pain; healing of skin ulcers.

Ozone is a toxic gas and it cannot be compared to either any usual immunological stimulus or to stable chemical compounds: firstly, nobody has ever described a cell receptor for ozone, and secondly the biochemical reactions with blood components generate various messengers with quite different half-lives, finalities and fate. Moreover, not only biological but also clinical responses have to be taken into account when using ozonetherapy in quite different pathologies such as cardiovascular, or autoimmune or orthopedic diseases. The hormetic dose response appears to be useful for describing the dual pharmacological response elicited by ozone, basically acting as a pro-drug. The most common form of the hormetic dose response curve, depicting low dose stimulatory and high dose inhibitory and toxic responses is the ß- or inverted U-shaped curve shown in Figure 3, panel a. However, the graphic illustration of the hormetic dose-response relationship between ozone and blood needs an explanation because it slightly differs from graphs presented on the effect of other stressors (Figure 3, panel b) [26,75-78]. It has been found that an ozone dose of only 10 µ;g/ml (0.21 μmol/ml) per ml of blood is fully neutralized by both uric acid and Aa, especially when the TAS of individual blood is between 1.5-1.9 mM [79]. It follows that the minimal reaction, if any, with PUFA will not generate enough messengers as ROS and LOP to trigger biological effects. In this case the small ozone dose is totally consumed by available free antioxidants and the ozonated blood will not display therapeutic activity. Gaseous ozone doses between 20 and 80 µ;g/ml (0.42-1.68 μmol/ml) per ml of blood are well calibrated against blood's TAS and both biological and therapeutic effects will ensue. A recent metabonomic study has shown that the blood antioxidant capacity is almost exhausted when the ozone dose has been raised to 160 µ;g/ml per ml of blood [74]. In simple words, too little ozone, unable to modify the homeostatic equilibrium, is unable to elicit the hormetic response. On the basis of the last observation, it would be most interesting to analyze the response in normal volunteers.

## Ozone Therapy in Oxidative-Stress Related Diseases

The metabolic syndrome is recognized as one of the most serious disease in Western countries caused by a number of metabolic alterations such as type-2 diabetes, hypercholesterolaemia, atherosclerosis and renal dysfunction with the common denominator represented by a chronic oxidative stress. Diabetic patients, particularly those with foot ulcers, are critical and today they still have a gloomy prognosis. This is because they need a multiform therapy aiming to eliminate the peripheral ischemia, the neuropathy and the infected skin lesions. The range of ozone concentrations between 15 and 35-50 µ;g/ml is safe also in individuals with a low TAS level and it appears to be particularly effective in PAD [43,80-85]. Several clinical studies performed in different hospitals seem to establish the validity of the inverted U-shaped curve in this frequent pathology (Figure 3, panel B). In line with "the concept of a beneficial effect within the context of a dose-response study is difficult to determine due to considerable biological complexity and the fact that beneficial effects are often seen with reference to a specific and relative setting" [17], a word of caution is necessary. This is especially true when ozone therapy is performed in different patients within the variety of three PAD's II, III and IV stages, according to the Leriche-Fontaine classification [86]. First of all it is necessary to trust the precision of ozone's dosages used by different clinicians and secondly, ozone activity cannot be compared with that expressed by a single compound (see, eg, Arsenic [76], and homocysteine [77]) in cultured cells. As it has been clarified, the real ozone messengers are H_2_O_2 _as a ROS and a variety of alkenals as LOP. These messengers act on different cells, have a quite different lifetime and alkenals are intrinsically toxic. Furthermore, each patient has his own medical history and his own psycho-physical reactivity. Consequently, ozone dosages between 0.42-0.84 µ;mol/ml generate less alkenals than dosages in the range 0.84-1.68 µ;mol/ml, and therefore patients with a low antioxidant capacity become more susceptible to a side effect like deep fatigue after the therapy session. Attention must be also paid to the type of pharmacological response achieved in different pathologies as either muscular-orthopedic or autoimmune diseases. So far, in the latter it remains unknown the ozone dosage, if any, able to increase the T-cell regulatory levels and activity. Consequently, at this stage the U-shaped curve remains meaningful only for PAD and only future trials will be able to define the ozone behavior in either stroke or chronic heart disease. Martinez-Sanchez *et al. *have also reported that the theoretical U-shaped curve fits the ozone therapy results [87]. Blood ozonation, even if performed within the therapeutic range and for a few minutes, represents always a calibrated acute oxidative stress. In order to never harm the patient, the strategy: "start low-go slow" is a golden rule to induce a valid adaptation to the far more dangerous chronic oxidative stress, typical of inflammatory and degenerative diseases [88]. Such an aspect implies that the final therapeutic effect is due to an average of progressively increasing ozone dosages.

The gas mixture medical grade oxygen-ozone can be proficiently used for the ozonation of blood because this incomparable liquid tissue contains an imposing array of antioxidants, which are able to tame not only its oxidant power but also its messengers (ROS and LOP) generated by the reactions with blood components. Therefore, if ozone is judiciously used within the established therapeutic window (0.42-1.68 μmol/ml per ml of autologous blood) in PAD, it can exert better therapeutic effects than the current therapy by prostacyclin analogue. Moreover, regarding the accompanying foot-related problems, both some ozone derivatives like ozonated water and different gradation of standardized ozonated vegetable oils will be used until complete healing [89,90]. As stroke, heart infarction and PAD are cumulatively the first cause of death and disability, if it will become possible to use ozone therapy in the public hospitals of the developed Countries, it may be possible to enter a phase where ozone will become an extensive remedy. Moreover, there is no doubt that either infective or autoimmune glomerulo-nephritis as well as end stages of renal failure associated with hemodialysis are characterized, to a different extent, by an imbalance between pro- and antioxidative mechanisms [91]. Moreover the kidney does not have the regenerative ability of liver and this is one of the reasons for explaining why too often "nephropaties lack a specific treatment and progress relentlessly to end-stage renal disease" [92]. Another important reason is that till today a valid strategy to reduce oxidative stress in renal diseases is not available. Ozone therapy, not only may correct a chronic oxidative stress, but it may also stimulate untapped resources able to afford some improvement [9,93]. It appears therefore reasonable to suggest the combination of conventional treatments with mild O_3_-AHT in any initial nephropathy for preventing the risk of progression towards a chronic disease.

In several Countries, among others Cuba, Russia, and Ukraine, treatments by ozone are already a reality, although different administration modalities, such as the infusion of ozonated saline and of the rectal insufflations of ozone, are in current use because inexpensive and applicable to thousands of patients every day [94]. Nevertheless, it is hoped that adequate ozone-based therapeutic treatments for patients affected by oxidative-stress related diseases could be implemented in every public hospital.

## Conclusions

During the last two decades the paradoxical behaviour of ozone has been clarified: when it is chronically inhaled, it is highly toxic for the pulmonary system because the enormous alveolar surface, unprotected by sufficient antioxidants, is exposed to the cumulative ozone dose, which causes a chronic inflammation. This is not surprising because even for oxygen [95], as well as for glucose and uric acid levels a modification of the physiological concentrations is deleterious.

On the basis of the mechanisms of action, ozone therapy appears to be a safe, economical, effective treatment for patients with cardiovascular disorders based on the following biological responses [26]:

a) it improves blood circulation and oxygen delivery to ischemic tissue owing to the concerted effect of NO and CO and an increase of intraerythrocytic 2,3-DPG level;

b) by improving oxygen delivery, it enhances the general metabolism;

c) it upregulates the cellular antioxidant enzymes and induces HO-1 and HSP-70;

d) it induces a mild activation of the immune system and enhances the release of growth factors from platelets;

e) it procures a surprising wellness in most of the patients, probably by stimulating the neuro-endocrine system. However, ozone dosages must be calibrated against the antioxidant capacity of the patient's plasma, or otherwise the "start low-go slow" strategy must be used evaluating the subjective feeling of the patient after each session.

It remains to be clarified whether some messengers present in the ozonated blood are able to stimulate the release of staminal cells in the patient's bone marrow.

The evaluation of results obtained in several clinical trials performed in PAD has allowed to establish that the dose-response relationship in PAD can be depicted as an inverted U-shaped hormetic model with a brief, initial lack of effect due to the potency of blood antioxidants. A mild acute oxidative stress induced by ozone in blood *ex vivo*, as several other mild stresses due to either heat or cold exposure, a transient ischemia, other chemicals and physical exercise are able to induce a sort of "preconditioning response" often leading to both a repair and an increased defense capacity well within the "overcompensation stimulation hormesis". This new achievement, added to an increasing wide consensus in carefully using gases as NO, CO, H_2_S, N_2_O and H_2 _as real medical drugs [68], suggests that also ozone may be soon included into this category. One of the basic functions of ozone, after dissolving in the water of plasma is to accelerate the exchange of protons and electrons or, in simple words, to reactivate the metabolism all over the body. In this way, crucial biological functions gone astray can recover indicating that ozone operated as both a biological response modifier and an antioxidant inducer.

It is hoped that this paper will elicit the interest of clinical scientists in evaluating ozone therapy in vascular, renal and diabetic diseases, thus translating the laboratory results to the patient's bed.

## Abbreviations

2,3-DPG: 2,3-diphosphoglycerate; 4-HHE: 4-hydroxy-2E-hexenal; 4-HNE: 4-hydroxy-2E-nonenal; Aa: ascorbic acid; ACTH: adrenocorticotropic hormone; ATP: adenosine triphosphate; CNS: central nervous system; EGF: epidermal growth factor; G6PD: glucose-6-phosphate dehydrogenase; GSH: glutathione; GSH-Rd: glutathione reductase; GSH-Px: glutathione peroxidase; GSH-Tr: glutathione transferase; GSSG: oxidized glutathione; HIV: human immunodeficiency virus; HO-1: heme-oxygenase-I; HSP-70: heat shock proteins (70 kDa); IFN-γ: interferon γ; IkB: inhibitor of NF-kB; LOAEL: lowest observed adverse effect level; LOP: lipid oxidation products; IL-8: interleukin 8; MDA: malondialdehyde; NADPH: nicotinamide adenine dinucleotide phosphate; NF-kB: nuclear factor kappa-light-chain-enhancer of activated B cells; NOAEL: no observable adverse effect level; OCSH: overcompensation stimulation hormesis; PaO_2_: partial pressure of arterial oxygen; PO_2_: partial pressure of oxygen; O_3_-AHT: ozonated autohemotherapy; PAD: peripheral arterial diseases; PDGF-B: platelet-derived growth factor, subunit B; PUFA: polyunsaturated fatty acids; ROS: reactive oxygen species; SOD: superoxide dismutase; TAS: total antioxidant status; TGF-β_1_: transforming growth factor β_1_; TNF-α: tumor necrosis factor.

## Competing interests

The authors declare that they have no competing interests.

## Authors' contributions

VAB and VT conceived, outlined the direction of, provided information to shape the manuscript content and discussion, gathered references, and drafted the manuscript. IZ refined the search for information, gathered references, and generated the figures. All authors have read and approved the final manuscript.

## Author Details

VAB, M.D., Emeritus professor of Physiology, Department of Physiology, University of Siena, Viale Aldo Moro, 2, 53100, Siena, Italy

IZ, in charge as post-doc position at the Department of Pharmaceutical Chemistry and Technology, Viale Aldo Moro, 2, 53100, Siena, Italy

VT, Associate professor in Pharmaceutical Technology and Chief of the Post-Graduate School of Hospital Pharmacy, University of Siena, Viale Aldo Moro, 2, 53100, Siena, Italy
